# Modified Calcanization of Tibia for Hindfoot Defect Reconstruction: Method and Preliminary Results

**DOI:** 10.1111/os.14214

**Published:** 2024-08-29

**Authors:** Rui Zhang, Xiaoyu Wang, Shenghe Liu, Shuo Qiu, Hongjiang Ruan, Qinglin Kang

**Affiliations:** ^1^ Department of Orthopedics Shanghai Sixth People's Hospital Affiliated to Shanghai Jiao Tong University School of Medicine Shanghai China

**Keywords:** Calcaneus Defect, Calcanization, Foot and Ankle, Function, Reconstruction

## Abstract

**Objectives:**

To introduce our modified technique for calcanization of the tibia in managing massive bony loss of hindfoot and preliminary outcomes.

**Methods:**

From January 2015 to December 2021, modified calcanization of the tibia were performed in 10 patients with unilateral loss of the calcaneus. Clinical outcomes were assessed based on the American Orthopaedic Foot & Ankle Society score and Symptom Checklist‐90‐Revised questionnaire. Paired two‐group *t*‐test was applied to compare the parameters.

**Results:**

The mean lengthened length of the tibia was 77.3 ± 3.0 mm (range, 74–83 mm). The mean external fixation time was 123.7 ± 52.1 days (range, 117–134 days) and the mean external fixation index was 1.601 ± 0.046 days/mm. All patients stuck to the postoperative follow‐up plan with an average follow‐up time of 29.7 ± 3.4 months (range, 24–35 months). Deformities of the injured limbs were well corrected. Based on American Orthopaedic Foot & Ankle Society score, eight good and two fair results were achieved. The mental status of all patients was within the normal range, and several indices of the Symptom Checklist‐90‐Revised questionnaire of each patient were improved after the whole procedure.

**Conclusion:**

We demonstrate that the modified calcanization of the tibia is qualified for total loss of calcaneus with limited complications. Early rehabilitation is attainable since external fixation time is shortened due to a simplified procedure.

## Graphical abstract

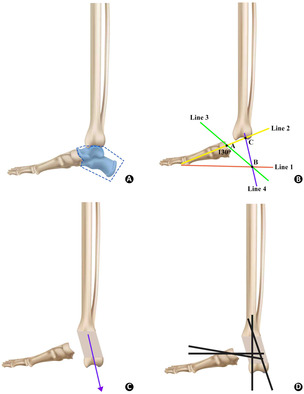

## Background

Hindfoot tissue defect remains complicated to manage due to the difficulties in restoring the wear‐resisting speciality of the heel as well as its characteristics of biomechanical conduction. This kind of lesion can be caused by either acute or chronic reasons, for example, landmine explosion, height falling accident, traffic accident, excessive debridement against hindfoot infection, and undue excision of hindfoot malignancies.^1^, ^2^, ^3^, ^4^, ^5^, ^6^ Unfortunately, there is no standard guideline for the management of hindfoot defect with massive bony loss, and the most common treatment for such abnormality is below‐knee amputation.^2^, ^3^, ^7^, ^8^

Advances in extremity lengthening and foot and ankle reconstructive surgeries have enabled staged reconstruction to become an alternative method to amputation. Calcanization of tibia was first introduced by Wardak et al.^3^ in 2008 to restore the bony structure of the hindfoot with massive bony loss. Through gradual lengthening, distal tibia and fibula, as well as adjacent soft tissue, could be distracted to occupy the place where once the tarsal bones, especially the calcaneus and talus, were situated, creating a robust support area for weight‐bearing. However, the direction of the elongation of this technique was primarily set along the mechanical axis of the tibia, which might lead to overload of the reconstructed hindfoot and result in pain and fatigue because they failed to restore the morphological features of the foot arch, the structural basis for normal weight bearing.^3^ Hence, we modified this technique through angulated elongation of the osteomized distal tibia and fibula to create a weight‐bearing point posterior to the frontal plane of the calf. We believe this modification could also somehow mimic the posterior node of the longitudinal arch of foot.

This study (i) introduces our modified technique for calcanization of the tibia in managing massive bony loss of hindfoot, (ii) reports preliminary outcomes in 10 patients who received such treatment, and (iii) summarizes current approaches for hindfoot reconstruction.

## Methods

After receiving institutional ethical approval (2023‐051‐(K)), participants who received the modified calcanization of the tibia for staged reconstruction of hindfoot defect between 2015 and 2021 at our institution were recruited according to the inclusion and exclusion criteria. The inclusion criteria for the study were a diagnosis of unilateral massive hindfoot structural loss, receiving our modified calcanization reconstruction surgery with other routine treatment, an age of over 16 years at the time of study, and follow‐up time over 2 years. The exclusion criteria included bilateral hindfoot defect, severe deformities other than the defect in the ipsilateral limb, orthopedic procedure within 12 months before last interview, and patients' refusal or intolerance of the reconstruction procedure. The present study eventually included 10 patients who received the modified reconstructive surgery. Demographic data were reviewed through hospital information system, which included the age at which the procedures were performed, gender, follow‐up time (FT), cause of the defect, side of injury, defect to reconstruction surgery interval (DRI), external fixation time (EFT), lengthened length (LL), and external fixation index (EFI).

### Surgical Technique

**FIGURE 1:**
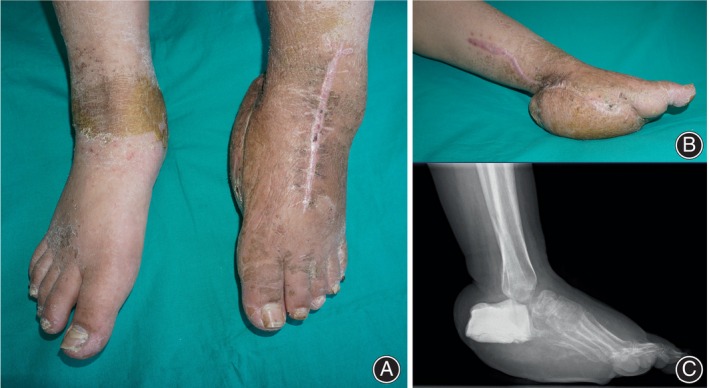
Preoperative appearance and radiography of a case with hindfoot composite defect. (A) Left foot and ankle was obviously shorter than the right ones. (B) Medial view of the defect foot after previous flap coverage of the hindfoot soft tissue defect. (C) The talus was removed in previous debridement and the calcaneus was filled with bone‐cement.

Patients who received such reconstructive surgery met the following indications: (1) complete loss of the calcaneus due to any reason, (2) intact distal facet of the talonavicular joint and intact forefoot for direction of the elongation, and (3) reliable skin and soft tissue coverage at hindfoot. The integrity of the talus and the malleolar joint was not necessary as these segments would be excised to enhance the smoothness of the bottom of the transported bone segments (Figure 1).

**FIGURE 2:**
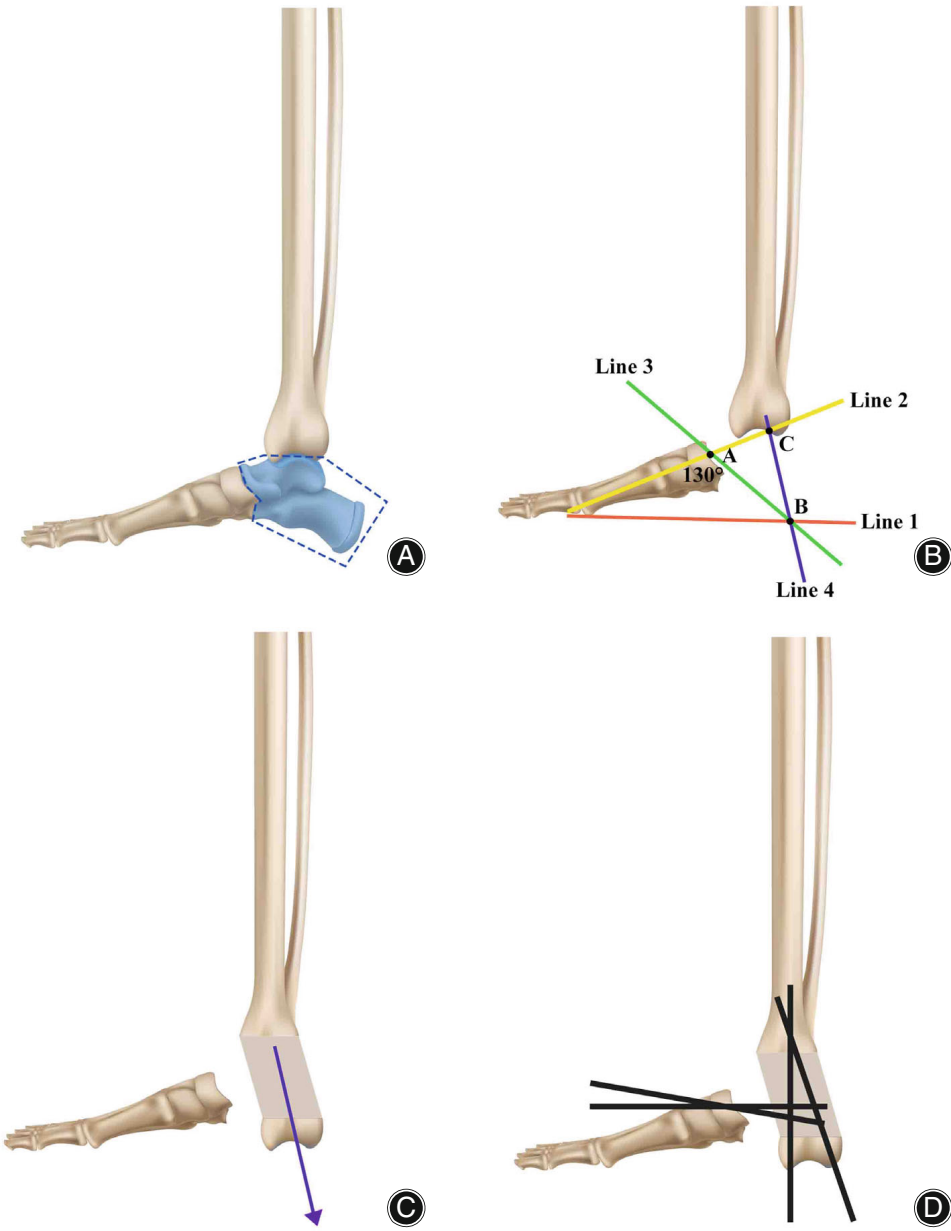
How the direction of tibial elongation was determined. (A) Indications for this technique are total loss of calcaneus with or without talus defect. The distal tibia, distal facet of the talonavicular joint and intact forefoot should be intact for direction of the elongation. (B) On the weight‐bearing lateral view of the foot with massive calcaneus defect, a horizontal line (line 1) was drawn tangent to the head of the first metatarsal bone; In the same plane, the anatomic axis of the first metatarsal bone (line 2) was drawn, which intersected with the distal facet of the talonavicular joint at point A. Through this very point, line 3 was drawn at 130° posterior to line 2 and it intersected with line 1 at point B. The posterior point of the trimmed malleolus (point C) was then connected to point B as line 4. (C) Distal tibia should be transported alongside line 4 until the reconstructed heel just surpassed line 1. (D) Kirschner wires were applied for the consolidation of the transported bone and for the fusion between the transported bone and the rest of the tarsal bones.

The Ilizarov apparatus was applied to the ipsilateral tibia and metatarsals of the defective side. Compared with the more detailed description of such a frame for calcanization, which was previously reported by Wardak et al.,^3^ we modified the elongation module, and prepared and installed it according to the following methods:

1. On the weight‐bearing lateral view of the foot with massive calcaneus defect, a horizontal line (line 1) was drawn tangent to the head of the first metatarsal bone;
2. In the same plane, the anatomic axis of the first metatarsal bone (line 2) was drawn, which intersected with the distal facet of the talonavicular joint at point A;
3. Through this very point, line 3 was drawn at 130° posterior to line 2 and it intersected with line 1 at point B;
4. The posterior point of the trimmed malleolus (point C) was then connected to point B as line 4;
5. The half ring of the elongation module should be perpendicular to the plane that was perpendicular to the sagital plane and went throught line 4 when installed to the segment to be transported and the proximal module was installed to the middle tibia, while another ring was installed to the proximal module in the plantar plane (Figure 2).

**FIGURE 3:**
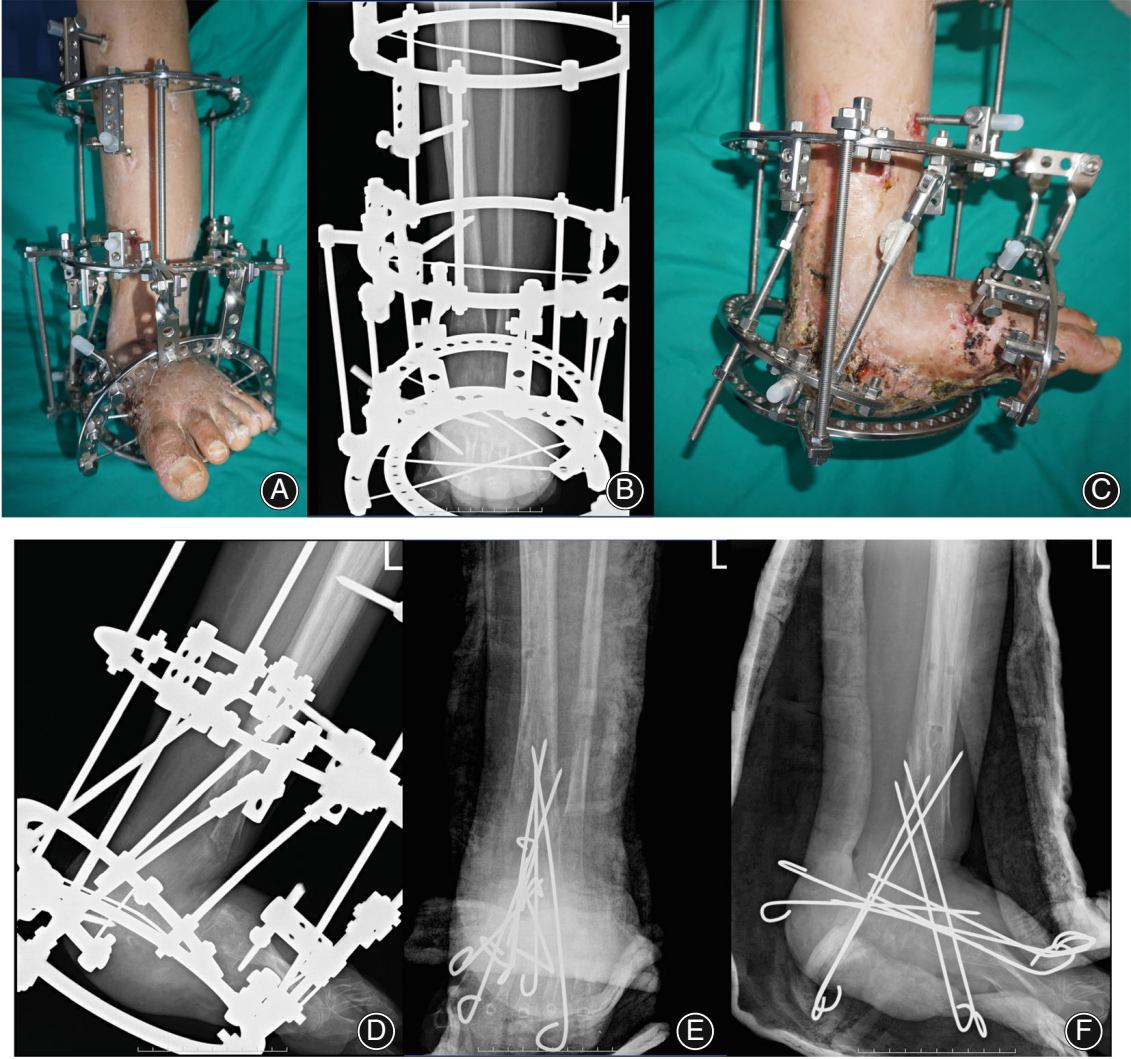
Appearance and radiography before and after external fixation was removed. Remnant of the calcaneus had been removed before the external fixation was applied. Supramalleolar osteotomy of the tibia and fibula was performed to distract the distal block toward the distal and posterior direction. (A, B) Anteroposterior view of the affected limb before the external fixation was removed. (C, D) Medial view of the affected limb before the external fixation was removed. Distraction osteogenesis was successful as the callus can be easily seen on radiographs. (E, F) Splint and Kirschner wires were applied after removal of the external fixation to enhance callus consolidation.

**FIGURE 4:**
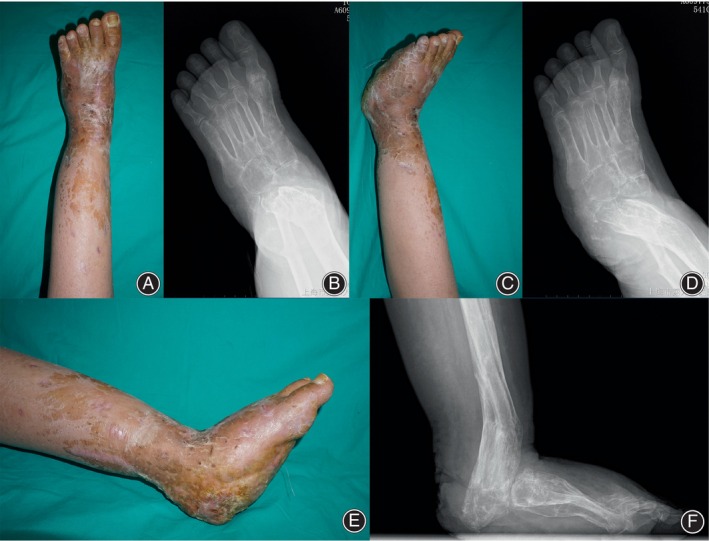
Postoperative appearance and radiography indicated satisfactory reconstruction of the defective foot and ankle. (A–D) The tibial‐calcaneal alignment was restored. (E, F) A more physiological pattern of the reproduced hindfoot bones guaranteed better weight‐bearing and ambulation.

After supramalleolar osteotomy and a latency of 10 days, the distal segments of the tibia and fibula were distracted distally and posteriorly until the reconstructed heel just surpassed the plantar ring. Acute correction was then performed to create a distance between the bisector lines of the proximal and distal segments of the lengthened tibia to mimic a normal tibial‐calcaneal distance (range 6–14 mm lateral). Kirschner wires were applied for the consolidation of the transported bone and for the fusion between the transported bone and the rest of the tarsal bones. A short‐leg splint was arranged in the first 6 weeks after removal of the external fixators (Figure 3). Kischner wires were then removed in steps (Figure 4).

### Functional Evaluation

Subjective satisfaction was reported using the 100‐mm visual analogue scale (VAS, 0 mm = totally unsatisfied, 100 mm = completely satisfied).^9^ Symptom Checklist‐90‐Revised (SCL‐90‐R), known as a brief self‐reported psychometric questionnaire, was also applied to evaluate patients' psychological situation.^10^ Outcomes were assessed using the American Orthopaedic Foot & Ankle Society (AOFAS) score as poor (0–50), fair (50–74), good (75–90) and excellent (90–100).^11^

### Statistical Analysis

GraphPad Prism 8.0 was applied for statistical analysis. Continuous variables were presented as *x¯* ± *s*. Paired two‐group *t*‐test was applied to compare VAS, SCL‐90‐R, and AOFAS previously and at last follow‐up. *p* value <0.05 was considered a significant difference.

## Results

### Demograpic Data

Five male patients and five female patients were included with an average age of 23.8 ± 4.6 years (range, 17–31 years). Four of them were injured due to falling accidents, five due to traffic accidents, and one due to calcaneal osteomyelitis. All lesions were unilateral. Soft tissue reconstruction was accomplished by a former surgical team with only hindfoot bony defect left. The mean DRI was 3.1 ± 1.3 months (range, 0–5 months). The mean LL was 77.3 ± 3.0 mm (range, 74–83 mm). The mean EFT and EFI was 123.7 ± 52.1 days (range, 117–134 days) and 1.601 ± 0.046 days/mm, respectively. All patients stuck to the postoperative follow‐up plan with an average FT of 29.7 ± 3.4 months (range, 24–35 months). Temporary pin track infection was observed in two patients and was treated with intravenous antibiotics (Table 1).

**Table 1:** Demographic data

Case	Age (years)	Gender	Side	Cause	DRI (mo)	FT (mo)	LL (mm)	EFT (d)	EFI (d/mm)	VAS‐pre	VAS‐post
1	17	Female	Left	FA	3	26	77	122	1.58	20	75
2	21	Male	Left	FA	4	24	83	128	1.54	27	80
3	26	Male	Left	FA	3	28	75	127	1.69	30	75
4	23	Male	Left	TA	3	31	76	121	1.59	30	80
5	30	Female	Left	FA	3	31	75	120	1.60	25	80
6	18	Male	Right	TA	3	30	77	127	1.65	31	75
7	22	Male	Right	TA	5	28	82	134	1.63	28	85
8	31	Female	Right	TA	4	35	74	117	1.58	36	90
9	25	Female	Right	TA	3	34	78	121	1.55	40	70
10	25	Female	Right	OM	0	30	76	120	1.58	35	70
Mean	23.8 ± 4.6				3.1 ± 1.3	29.7 ± 3.4	77.3 ± 3.0	123.7 ± 5.1	1.601 ± 0.046	30.2 ± 5.8	78.0 ± 6.3

Abbreviations: d, day; DRI, defect to reconstruction surgery interval; EFI, external fixation index; EFT, external fixation time; FA, falling accident; FT, follow‐up time; mm, milimeter; mo, month; OM, osteomyelitis; TA, traffic accident; VAS, visual analogue scale.

### Functional Outcomes

Subjective evaluation according to VAS (30.2 ± 5.8 points preoperatively, 78.0 ± 6.3 points at last follow‐up, *p* < 0.001) was significantly improved and all patients were satisfied with the eventual results (Table 1).

Detailed AOFAS data are shown in Table 2. The total AOFAS was significantly improved (25.0 ± 6.3 points preoperatively, 81.5 ± 6.9 points at last follow‐up, *p* < 0.05) and there were eight good and two fair results, respectively. Nine of our patients gained stable lower extremities and could walk over six blocks without any aid. Slight pain and claudication could be observed temporarily but these symptoms did not disturb their daily activities. Two patients reported having difficulty in walking over uneven surfaces.

**Table 2:** Detailed AOFAS evaluated preoperatively and at last interview.

Case (preoperative/last interview)	Pain	ALSR	MWDB	Walking surfaces	Gait abnormality	Sagital motion	Hindfoot motion	AHS	Alignment	Total
1	20/40	0/10	0/5	0/3	0/8	4/0	3/0	0/8	0/10	27/84
2	10/40	0/10	0/5	0/3	0/4	4/0	3/0	0/8	0/10	17/80
3	20/40	0/10	0/5	0/5	0/8	4/0	3/0	0/8	0/10	27/86
4	20/40	0/10	0/5	0/5	0/8	4/0	3/0	0/8	0/10	27/86
5	20/40	0/10	0/5	0/5	0/8	4/0	3/0	0/8	0/10	27/86
6	10/30	0/10	0/5	0/3	0/8	4/0	3/0	0/8	0/10	17/74
7	20/40	0/10	0/5	0/3	0/8	4/0	3/0	0/8	0/10	27/84
8	30/40	0/10	0/5	0/5	0/8	4/0	3/0	0/8	0/10	37/86
9	20/40	0/10	0/5	0/3	0/8	4/0	3/0	0/8	0/10	27/84
10	10/30	0/10	0/5	0/3	0/4	4/0	3/0	0/8	0/5	17/65
Preoperative	18.0 ± 6.3	0.0 ± 0.0	0.0 ± 0.0	0.0 ± 0.0	0.0 ± 0.0	4.0 ± 0.0	3.0 ± 0.0	0.0 ± 0.0	0.0 ± 0.0	25.0 ± 6.3
Last interview	38.0 ± 4.2	10.0 ± 0.0	5.0 ± 0.0	3.8 ± 1.0	7.2 ± 1.7	0.0 ± 0.0	0.0 ± 0.0	8.0 ± 0.0	9.5 ± 1.6	81.5 ± 6.9
*t* value	13.42	/	/	11.64	13.50	/	/	/	19.00	38.83
*p* value	<0.001	/	/	<0.001	<0.001	/	/	/	<0.001	<0.001

Abbreviations: AHS, ankle‐hindfoot stability; ALSR, activity limitations and support requirement; AOFAS, American Orthopaedic Foot & Ankle Society score; MWDB, maximum walking distance, blocks.

The mental status of all patients was within the normal range, and interestingly, several indices of SCL‐90‐R of each patient were improved after the whole procedure, which indicated the salvage of the limb might also have contributed to the stabilization of mental status (Table 3).

**Table 3:** Psychological status assessed with the SCL‐90‐R questionnaire.*

Case	Phobia T1/T2/T3/T4	Anxiety T1/T2/T3/T4	Depression T1/T2/T3/T4	Somatization T1/T2/T3/T4	Obsessive‐compulsive T1/T2/T3/T4	Sensitivity T1/T2/T3/T4	Hostility T1/T2/T3/T4	Insomnia T1/T2/T3/T4	Psychoneuroticism T1/T2/T3/T4
1	1.3/1.2/1.1/1.1	1.4/1.2/1.1/1.1	1.3/1.2/1.2/1.1	1.2/1.1/1.1/1.0	1.0/1.0/1.0/1.0	1.5/1.3/1.3/1.1	1.1/1.1/1.1/1.1	1.2/1.2/1.1/1.0	1.2/1.1/1.1/1.0
2	1.2/1.2/1.1/1.1	1.3/1.2/1.1/1.1	1.3/1.2/1.2/1.0	1.2/1.1/1.0/1.0	1.0/1.0/1.0/1.0	1.4/1.2/1.2/1.1	1.1/1.1/1.1/1.1	1.3/1.2/1.1/1.0	1.2/1.1/1.1/1.0
3	1.1/1.2/1.1/1.1	1.4/1.2/1.1/1.1	1.3/1.2/1.1/1.1	1.2/1.1/1.1/1.0	1.0/1.0/1.0/1.0	1.4/1.1/1.1/1.1	1.0/1.1/1.1/1.0	1.1/1.1/1.1/1.0	1.1/1.1/1.1/1.0
4	1.1/1.1/1.1/12	1.2/1.1/1.1/1.1	1.4/1.3/1.2/1.0	1.2/1.1/1.1/1.0	1.0/1.0/1.0/1.0	1.2/1.1/1.1/1.1	1.0/1.1/1.0/1.0	1.0/1.1/1.1/1.0	1.1/1.1/1.1/1.0
5	1.3/1.0/1.1/1.1	1.3/1.2/1.2/1.0	1.2/1.1/1.1/1.0	1.2/1.1/1.1/1.0	1.0/1.0/1.0/1.0	1.2/1.1/1.1/1.1	1.0/1.1/1.0/1.0	1.3/1.1/1.1/1.0	1.2/1.1/1.1/1.0
6	1.2/1.2/1.1/1.0	1.1/1.1/1.1/1.0	1.3/1.1/1.1/1.0	1.1/1.1/1.0/1.0	1.0/1.0/1.0/1.0	1.3/1.1/1.1/1.1	1.0/1.0/1.0/1.0	1.3/1.0/1.1/1.0	1.2/1.1/1.1/1.1
7	1.2/1.1/1.1/1.0	1.2/1.2/1.1/1.0	1.1/1.1/1.1/1.1	1.1/1.1/1.0/1.0	1.0/1.0/1.0/1.0	1.1/1.1/1.3/1.1	1.0/1.0/1.0/1.1	1.3/1.0/1.1/1.0	1.1/1.1/1.1/1.0
8	1.3/1.1/1.0/1.0	1.4/1.2/1.0/1.0	1.2/1.2/1.1/1.0	1.2/1.1/1.1/1.0	1.1/1.0/1.0/1.0	1.3/1.1/1.2/1.0	1.1/1.0/1.0/1.0	1.0/1.0/1.1/1.0	1.0/1.0/1.1/1.0
9	1.3/1.2/1.1/1.0	1.4/1.2/1.0/1.1	1.3/1.3/1.1/1.1	1.1/1.1/1.0/1.1	1.0/1.0/1.0/1.0	1.1/1.1/1.1/1.0	1.1/1.1/1.0/1.0	1.1/1.2/1.0/1.0	1.0/1.0/1.1/1.1
10	1.2/1.1/1.1/1	1.2/1.2/1.1/1.0	1.3/1.2/1.0/1.1	1.1/1.1/1.0/1.0	1.0/1.0/1.0/1.0	1.3/1.2/1.0/1.0	1.0/1.0/1.0/1.0	1.3/1.1/1.0/1.0	1.1/1.1/1.1/1.0
Mean	1.22/1.14/1.09/1.06	1.29/1.18/1.09/1.05	1.27/1.20/1.12/1.05	1.16/1.10/1.05/1.01	1.01/1.00/1.00/1.00	1.28/1.14/1.14/1.07	1.04/1.06/1.03/1.03	1.20/1.10/1.08/1.00	1.12/1.08/1.10/1.02
*p*1/*p*2/*p*3	0.053/0.002*/0.003*	0.003*/0.002*/<0.001*	0.010*/<0.001*/<0.001*	0.005*/<0.001*/<0.001*	0.343/0.343/0.343	0.001*/0.022*/<0.001*	0.591/0.037*/0.037*	0.063/0.013*/<0.001*	0.037*/0.443/0.009*

* T1, 2 days before external fixation; T2, 10 days after external fixation; T3, the day external fixation replaced by internal fixation; T4, 2 months after internal fixation was removed. *p*1, *p* value between T1 and T2; *p*2, *p* value between T1 and T3; *p*3, *p* value between T1 and T4.

## Discussion

The most important modification of our calcanization technique is that the direction of elongation was calculated according to the preoperative radiographic results. Under this calculation, the distracted callus will eventually mimic a normal tibial‐calcaneal distance on the anteroposterior view, and also mimic a normal longitudinal foot arch on lateral view. Hence, the appearance of the regenerated hindfoot would share similarity with a normal heel. Besides, this modification gives a more physiological framework.

This study clinically identified that this modified calcanization technique generally offers patients with complete calcaneus and talus loss an valuable opportunity for limb salvage. According to our preliminary outcomes, these patients regained ankle‐hindfoot stability and could finally walk through blocks without aid.

### Summary of other Techniques for Hindfoot Reconstruction and Comparison

Complex defect of hindfoot still remains challenging, for which the available treatment options lack proper definition due to the rarity of this kind of injury. Aside from emergency amputation, suggested limb‐salvaging plan for such complicated trauma in the early literature included flap coverage combined with delayed internal fixation, malleolar arthrodesis, or osteosynthesis with only Kirschner wires.^12^, ^13^, ^14^, ^15^, ^16^ However, these attempts result in unsatisfactory outcomes and secondary calf amputation because of multiple complications including osteomyelitis.^17^, ^18^

Preservation of such limbs remains controversial among traumatologists. Although primary amputation offers early rehabilitation as well as reduced hospitalization, amputation is still definitely a tough decision for the patients with lifelong disability and prosthesis maintenance fee.^18^, ^19^ Recent decades have witnessed novel solutions for complex hindfoot defect with various success rates, which included osteofasciocutaneous flap surgery,^20^, ^21^, ^22^, ^23^, ^24^, ^25^, ^26^, ^27^, ^28^, ^29^ allograft with autologous graft or bioactive components for induced osteogenesis,^4^, ^30^, ^31^, ^32^, ^33^, ^34^, ^35^, ^36^, ^37^, ^38^ and alloy substitution.^7^, ^39^, ^40^, ^41^ Drawbacks of these approaches lie in donor site morbidity, difficulties in anastomosis, foreign body rejection, long‐term bone resorption and fracture, material fatigue, and infection.

The Ilizarov technique has been shown to be capable of providing painless, sensible, and plantigrade foot with applausive outcomes in treating different degrees of hindfoot defect. Partial traumatic defect within 35% of the calcaneal tuberosity has been reported to be effectively restored with acute or gradual lengthening using Ilizarov external fixators.^1^, ^5^

For complete loss of the calcaneus, tibial longitudinal elongation with secondary malleolar arthrodesis, also known as calcanization of the tibia, was first reported by Wardak et al.^3^ in 2008. Twenty‐six patients in their trial received satisfactory results in the aspects of painless plantigrade foot, stable ambulation, and minor claudication, although most of them had difficulty in walking over uneven surface due to ankle fusion. Since then, Mekki et al.^2^ demonstrated their two‐staged elongation with Ilizarov external fixators for hindfoot reconstruction after fusion of the tibia and talus in patients who suffered from calcaneal osteomyeolitis and received calcaneoectomy. First‐stage vertical elongation of the tibia creates enough height at hindfoot, while second‐stage posterior transport of the talus block imitates the posterior point of the foot arch, which optimized the physiological weight‐bearing pattern of the reconstructed limb, compared with the vertical transport of the tibia using the conventional calcanization procedure. Despite the fact that all limbs were eventually anatomically stable and well‐functioning with minor complications, two‐staged correction required prolonged time in frame (at least 8 months), which would bring about inconvenience and delayed weight‐bearing of the affected limb. Hence, we modified these two approaches by orienting the only elongation procedure according to the anatomic features of the individual feet. This modification not only provided limb salvage and anatomically physiological weight‐bearing characteristics, which we believed to reduce difficulty in walking over uneven surfaces, but also reduced external fixation time for rehabilitation as early as possible. Through this direct elongation, a mean of only 123.7 days of external fixation was observed with acceptable outcomes including smooth walking without aid, and reduced negative emotions due to abnormality. Although lacking in histological evidence, the patients regained capability of hindfoot weight‐bearing and walking function of the involved limb, indicating that the connection/fusion between the regenerated calcaneus and midfoot bones, either fibrous or bony, was robust and tight.

### Limitations and Difficulties

While this modified Ilizarov technique does offer an option for limb salvage with massive hindfoot defect, it shares shortcomings with other studies, such as wire infection and a risk of neurovascular pulling injury during distraction.^2^, ^3^ On the other hand, in order to make precise calculations for the direction of elongation, it is of vital importance to obtain highly qualified preoperative fluoroscopic data, which could be rather difficult for those underdeveloped centers. It is also challenging for orthopedists to avoid iatrogenic damage to certain vessels and nerves during external frame installation, especially a proemial pedicled or free flap was performed to cover the hindfoot wound. More related studies with a larger sample size and longer follow‐up time will reveal the efficacy of this modified technique in hindfoot reconstruction.

### Prospects of Clinical Application

Occasional traffic accidents as well as regional armed conflicts are still threatening the integrity of the hindfoot of those who are engaged in the accidents and armed conflicts. Our technique would be a prospective approach for hindfoot reconstruction under the circumstances in which the calcaneus and the talus are lost acutely or chronically. However, one of the most important preconditions for the application of this technique is that the involved hindfoot should be covered with reliable soft tissue in advance. With deeper understanding of lower limb anastosomes and with the combination of flap surgery, the modified calcanization technique will certainly be a powerful tool for multiple tissue defect reconstruction of the heel.

## Conclusion

We have demonstrated that this modified calcanization of the tibia is qualified for total loss of the calcaneus with limited complications. Early rehabilitation becomes attainable since external fixation time is shortened due to the simplified procedure. Application of such a technique will be advantageous to patients experiencing massive hindfoot defect due to battlefield, traffic, and height‐falling injuries. Hence, patients and surgeons must weigh the lifelong quality of life after amputation against multiple‐staged surgical intervention for limb salvage before selecting a proper treatment for hindfoot reconstruction.

## Author contributions

All authors contributed to the creation of this article. Rui Zhang and Xiaoyu Wang contributed equally to this article as co‐first authors. Authors contributed separately as follows. Concept/idea/research design: Hongjiang Ruan and Qinglin Kang. Acquisition of data: Rui Zhang and Xiaoyu Wang. Analysis and interpretation of data: Xiaoyu Wang and Shenghe Liu. Writing/review/editing of manuscript: Rui Zhang and Xiaoyu Wang. Final approval of the manuscript: Hongjiang Ruan and Qinglin Kang. Acquisition of funding: Qinglin Kang. Providing subjects: Qinglin Kang and Shuo Qiu.

## Conflict of Interest

All authors claim no financial disclosure or any conflict of interest.

## Ethics Statement

Statement of Institutional Review Board or Ethics Committee approval of the study protocol: The study protocol conforms to the ethical guidelines of the 1975 Declaration of Helsinki as reflected in *a priori* approval by Shanghai Sixth People's Hospital Ethics Committee (No. 2023‐KY‐051(k)). Name of the public trials registry and the registration number: Chinese Clinical Trial Registry (ChiCTR2300072615).

## Funding Information

This work was supported by the National Natural Science Foundation of China (82072421) and Natural Science Foundation of Shanghai (20ZR1442200).
